# Advanced gastrointestinal stromal tumor: reliable classification of imatinib plasma trough concentration via machine learning

**DOI:** 10.1186/s12885-024-11930-6

**Published:** 2024-02-24

**Authors:** Pan Ran, Tao Tan, Jinjin Li, Hao Yang, Juan Li, Jun Zhang

**Affiliations:** 1 Department of Gastrointestinal Surgery, the First Affiliated Hospital of Chongqing Medical University, Chongqing, 400016 China; 2 Department of Internal Medicine, Chongqing Key Laboratory of Translational Research for Cancer Metastasis and Individualized Treatment, Chongqing University Cancer Hospital, Chongqing, 400030 China; 3 Department of Pharmacy, the First Affiliated Hospital of Chongqing Medical University, Chongqing, 400016 China

**Keywords:** Gastrointestinal stromal tumor, Imatinib, Therapeutic drug monitoring, Machine learning, Plasma trough concentration

## Abstract

**Aim:**

Patients with advanced gastrointestinal stromal tumors (GISTs) exhibiting an imatinib plasma trough concentration (IM C_min_) under 1100 ng/ml may show a reduced drug response rate, leading to the suggestion of monitoring for IM C_min_. Consequently, the objective of this research was to create a customized IM C_min_ classification model for patients with advanced GISTs from China.

**Methods:**

Initial data and laboratory indicators from patients with advanced GISTs were gathered, and the above information was segmented into a training set, validation set, and testing set in a 6:2:2 ratio. Key variables associated with IM C_min_ were identified to construct the classification model using the least absolute shrinkage and selection operator (LASSO) regression and forward stepwise binary logistic regression. Within the training and validation sets, nine ML classification models were constructed via the resampling method and underwent comparison through the Brier scores, the areas under the receiver-operating characteristic curve (AUROC), the decision curve, and the precision-recall (AUPR) curve to determine the most suitable model for this dataset. Two methods of internal validation were used to assess the most suitable model's classification performance: tenfold cross-validation and random split-sample validation (test set), and the value of the test set AUROC was used to evaluate the model's classification performance.

**Results:**

Six key variables (gender, daily IM dose, metastatic site, red blood cell count, platelet count, and percentage of neutrophils) were ultimately selected to construct the classification model. In the validation set, it is found by comparison that the Extreme Gradient Boosting (XGBoost) model has the largest AUROC, the lowest Brier score, the largest area under the decision curve, and the largest AUPR value. Furthermore, as evaluated via internal verification, it also performed well in the test set (AUROC = 0.725).

**Conclusion:**

For patients with advanced GISTs who receive IM, initial data and laboratory indicators could be used to accurately estimate whether the IM C_min_ is below 1100 ng/ml. The XGBoost model may stand a chance to assist clinicians in directing the administration of IM.

## Introduction

Gastrointestinal stromal tumors (GISTs) are the most common mesenchymal tumors of the digestive tract [1]. Acquired functional mutations in the tyrosine-protein kinase growth factor receptor proto-oncogene (KIT) and platelet-derived growth factor-alpha gene lead to increased tyrosine kinase activity, which is considered a key factor in the pathogenesis of GIST [1–3]. Imatinib (IM), a tyrosine kinase inhibitor (TKI), blocks KIT receptor activity and has become the conventional first-line therapy for patients with advanced GISTs [4], which inhibits proliferation and promotes apoptosis of GIST cells [4–6]. Therefore, the IM plasma trough concentration (C_min_) is intimately linked to the effectiveness of treatment [7].

The IM C_min_ of patients with advanced GISTs below 1100 ng/mL showed a shorter time to progression, according to a prior study by Demetri et al. [8]. Meanwhile, marked inter-individual variability in IM pharmacokinetics between subjects has been observed [9–11], leading to the suggestion of monitoring for IM C_min_ [12]. However, the absence of a therapeutic drug monitoring (TDM) platform in certain hospitals is due to restricted health conditions, making the sampling and examination of TDM for IM expensive both temporally and financially. Thus, there is a need for more convenient concentration classification tools than TDM, such as rapidly developing machine learning (ML) methods [13, 14], which can provide a reference for clinicians to make clinical decisions, thus reducing the cost of time and money for patients.

ML has an irreplaceable position in data analysis and can help promote data-driven estimation when predicting from multiple variables and capturing non-linear variable relations to construct a model with high classification performance [15, 16]. Therefore, this study aimed to streamline the process of IM C_min_ monitoring using the ML model based on patients’ initial data (demographic, treatment, and clinical information) and laboratory indicators.

## Materials and methods

### Patients and data

**Fig. 1:**
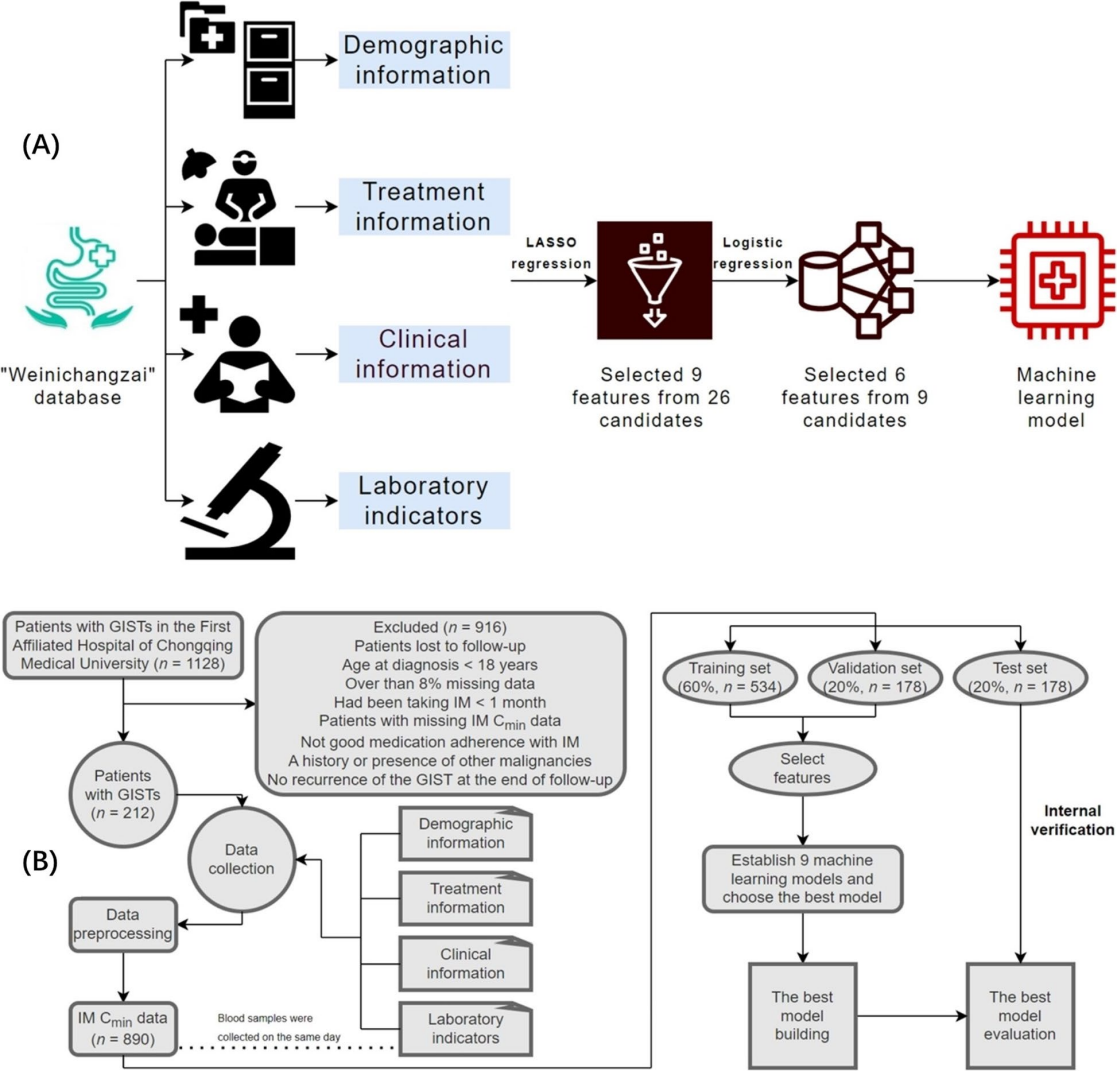
The case screening flowchart and the schematic representation of the study design. **A** This figure showed how the data were gathered from the “Weinichangzai” database of the First Affiliated Hospital of CMU, and all variables included demographic information, treatment information, clinical information, and laboratory indicators. There were 26 feature variables collected, and 6 key variables related to IM C_min_ were screened using the LASSO regression and binary logistic regression. Moreover, the study used the 6 key variables to establish a classification model. **B** The flowchart of study design. Abbreviations: GIST, gastrointestinal stromal tumor; CMU, Chongqing Medical University; IM, imatinib; C_min_, plasma trough concentration; LASSO, least absolute shrinkage and selection operator.

Demographic information of patients with advanced GISTs who were followed up at the First Affiliated Hospital of Chongqing Medical University (CMU) between January 2000 and August 2023 was gathered retrospectively. Meanwhile, IM C_min_ data, treatment information, clinical information, and laboratory indicators were collected in the same patient with advanced GIST from April 2017 to August 2023. For patients with advanced GISTs, our team generally recommends that patients go to the GIST specialist clinic for follow-up every 3 or 6 months or so for an abdomen ultrasound or CT examination, to observe the tumor situation and monitor the IM C_min_ simultaneously. It is worth noting that blood samples were collected and separated for routine blood, liver, and kidney function examinations from patients with GISTs on the same day the venous blood samples were collected to determine IM C_min_. The inclusion criteria were as follows: (1) verification of GIST through biopsy or postoperative pathology, (2) age over 18 years, (3) good medication adherence with IM, (4) less than 8% missing data, and (5) had been taking IM ≥ 1 month. The exclusion criteria were as follows: (1) patients with GISTs who had undergone complete tumor resection and had no recurrence of the tumor at the end of follow-up, (2) history or existence of other malignancies, (3) patients with missing IM C_min_ data, and (4) patients lost to follow-up. The case screening flowchart and the schematic representation of the study design are displayed in Fig. 1.

We established a database called “Weinichangzai”, which included initial data for each patient, and all patients’ initial data was obtained through the GIST specialist outpatient clinic, telephone calls, WeChat, and other interaction tools. Initial data included demographic information (including age at diagnosis, age at blood sampling, gender, and residence); treatment information (including surgical procedures (1, gastrectomy; 2, non-gastric operation) [17, 18] and daily IM dose); and clinical information (including expression of DOG-1/CD117/CD34, metastatic site (1, liver; 2, non-liver) [17, 18], and primary tumor site).

### Determination of IM C_min_

All patients with advanced GISTs were advised to take IM at lunchtime, and a 3 ml venous blood sample was collected in a heparinized vial on the second day (generally 24 ± 3 h following the previous dose) before taking an IM dose. Blood samples were centrifuged at room temperature for 5 min at 3000 g. A protocol was established for determining IM C_min_ according to the method described by Tan et al. [19] and Roth et al. [20]. Plasma samples were pretreated by protein precipitation. We added 100 μl of acetonitrile and 50 μl (50%) of perchloric acid successively to plasma (0.5 ml), swirled the solution for 20 s, and centrifuged it at 10,800 rpm for 15 min. We added 50 μl of neutralizing solution (containing 1.4 g potassium carbonate and 0.65 g potassium chloride dissolved in 5 ml of purified water) to 400 μl of the supernatant, and the mixture was vortexed thoroughly before submerging for 30 min at 4 °C. Thirty microliters of the supernatant was injected into a high-performance liquid chromatography system. The lower limit of quantification was set at 50 ng/ml.

### Laboratory indicators

Blood samples were collected from patients with advanced GISTs, and separated for routine blood, liver, and kidney function examinations. Routine blood examinations included the white blood cell count (WBC), platelet count (PLT), percentage of neutrophils (NEU%), red blood cell count (RBC), hemoglobin (HB), and percentage of lymphocytes (LYM%). Liver function examinations included alanine aminotransferase (ALT), aspartate aminotransferase (AST), total bilirubin (TBIL), direct bilirubin (DBIL), indirect bilirubin (IBIL), gamma-glutamyl transpeptidase (GGT), and alkaline phosphatase (AKP). Kidney function examinations included creatinine (Cr), urea nitrogen (BUN), and the estimated glomerular filtration rate (eGFR).

### Method of feature selection

We collected 26 candidates from demographic information, treatment information, clinical information, and laboratory indicators. To obtain the best predictive performance, the variable selection was performed on 26 candidates using the LASSO (least absolute shrinkage and selection operator) regression with tenfold cross-validation, which could compress the variable coefficients to prevent overfitting and solve severe collinearity problems [21, 22]. LASSO regression analyses were performed using “Extreme Smart Analysis” (www.xsmartanalysis.com). To further control the influence of confounding factors, variables selected by LASSO regression were analyzed by forward stepwise binary logistic regression (LR) to obtain key variables. LR analyses were performed using SPSS version 27.0 (IBM Corp, Armonk, NY, USA).

### Selection method of ML algorithm

In this study, we randomly divided the dataset into three sets: the training (60%) and validation sets (20%) for ML model development and the test set (20%) for performance evaluation. The randomization's success was determined by comparing baseline characteristics in each group. Nine types of ML algorithms were used to construct the classification models in this study: Extreme Gradient Boosting (XGBoost), Light Gradient Boosting Machine (LightGBM), Random Forest (RF), Gaussian Naive Bayes (GNB), Complement Naive Bayes (CNB), Multilayer Perceptron (MLP), Support Vector Machine (SVM), K-Nearest Neighbour (KNN), and Adaptive Boost (AdaBoost). All analyses were performed using “Extreme Smart Analysis”, which can also select the best-performing hyper-parameters using the grid-search method.

Within the training and validation sets, nine ML classification models were constructed via the resampling method and underwent comparison through the Brier scores, the areas under the receiver-operating characteristic curve (AUROC), the decision curve, and the precision-recall (AUPR) curve to determine the most suitable model for this dataset, which were important indicators that can be used to evaluate classification models. Two methods of internal validation were used to assess the most suitable model's classification performance: tenfold cross-validation and random split-sample validation (test set). To explain the model predictions, we used Shapley Additive Explanations (SHAP) of “Extreme Smart Analysis” to calculate the Shapley values of the test set. SHAP values are based on Shapley values in cooperative game theory to proceed with the best explanation of the output of our machine-learning model [23].

### Statistical analysis

Continuous variables (non-normal distribution) are described using median and interquartile range (IQR) values, and categorical variables are presented as frequencies (percentages). The Mann–Whitney U-test (non-normal distribution) was used to assess the differences in continuous variables between the training, validation, and test sets. Categorical variables were compared between the training, validation, and test sets using the Pearson chi-square test, and Fisher exact test. Statistical significance was set at *p* < 0.05. All P values were calculated as two-tailed. All analyses were performed using SPSS version 27.0 (IBM Corp, Armonk, NY, USA).

## Results

### Baseline characteristics

In total, 212 patients with advanced GISTs, based on the inclusion and exclusion criteria, were included, of whom 890 IM C_min_ data were collected. Missing data were filled by imputing the data via the RF algorithm [24]. The mean value of IM C_min_, the label variable, was 1469.59 ng/mL, with a standard deviation (SD) value of 755.71 ng/ml. In this dataset, 31.24% of IM C_min_ values were < 1100 ng/ml. More than half were males (59.10%). The mean age at diagnosis was 56 years, and 18.54% of this dataset underwent gastrectomy. The daily IM dose in this dataset (76.29%) was 400 mg/day. The comparison of baseline characteristics between the test set (20%) and training and validation sets (80%) is shown in Table 1, without any statistically significant differences in the variables between the two groups (*p* > 0.05). The comparison of baseline characteristics between “IM C_min_ ≤ 1100 ng/ml” and “IM C_min_ > 1100 ng/ml” was shown in Table 2, significant differences were observed between the groups according to age at diagnosis, age at blood sampling, gender, daily IM dose, metastatic site, NEU%, RBC, HB, LYM%, ALT, TBIL, IBIL, GGT, Cr, BUN, and eGFR (*p* < 0.05).

**Table 1:** Baseline characteristics of 890 IM C_min_ data from 212 patients with advanced GISTs

Categories	Variables	All	Training and Validation Sets (80%)	Test Set	*P*
				(20%)	
		(***n*** = 890)	(***n*** = 712)	(***n*** = 178)	
Demographic information	Age at diagnosis (years)	56 (49–65)	56 (49–65)	58 (50–65)	0.430^a^
	Age at blood sampling (years)	61 (53–68)	61 (53–68)	61 (53–67)	0.805^a^
	Gender				0.708^b^
	Male	526 (59.10%)	423 (59.41%)	103 (57.87%)	
	Female	364 (40.90%)	289 (40.59%)	75 (42.13%)	
Treatment information	Surgical procedure				0.666^b^
	Gastrectomy	165 (18.54%)	130 (18.26%)	35 (19.66%)	
	Non-gastric operation	725 (81.46%)	582 (81.74%)	143 (80.34%)	
	Daily IM dose				0.324^b^
	≤ 200mg/d	28 (3.15%)	19 (2.67%)	9 (5.06%)	
	300mg/d	89 (10.00%)	73 (10.25%)	16 (8.99%)	
	400mg/d	679 (76.29%)	546 (76.69%)	133 (74.72%)	
	500mg/d	54 (6.07%)	45 (6.32%)	9 (5.06%)	
	≥ 600mg/d	40 (4.49%)	29 (4.07%)	11 (6.18%)	
Clinical information	DOG-1				0.144^b^
	Positive	859 (96.52%)	684 (96.07%)	175 (98.31%)	
	Negative	31 (3.48%)	28 (3.93%)	3 (1.69%)	
	CD117				0.771^b^
	Positive	878 (98.65%)	702 (98.60%)	176 (98.88%)	
	Negative	12 (1.35%)	10 (1.40%)	2 (1.12%)	
	CD34				1.000^b^
	Positive	795 (89.33%)	636 (89.33%)	159 (89.33%)	
	Negative	95 (10.67%)	76 (10.67%)	19 (10.67%)	
	Primary tumor site				0.617^b^
	Stomach	293 (32.92%)	239 (33.57%)	54 (30.34%)	
	Small intestine	352 (39.55%)	276 (38.76%)	76 (42.70%)	
	Colorectum	74 (8.31%)	62 (8.71%)	12 (6.74%)	
	Other	171 (19.21%)	135 (18.96%)	36 (20.22%)	
	Metastatic site				1.000^b^
	Liver	405 (45.51%)	324 (45.51%)	81 (45.51%)	
	Non-liver	485 (54.49%)	388 (54.49%)	97 (54.49%)	
Laboratory indicators	WBC (*10^9^/L)	4.32 (3.47–5.39)	4.33 (3.48–5.37)	4.30 (3.46–5.53)	0.852^a^
	PLT (*10^9^/L)	173 (136–220)	173 (135–217)	178 (137–223)	0.484^a^
	NEU%	61.25 (53.30–68.90)	61.40 (53.43–69.25)	60.65 (52.05–68.63)	0.422^a^
	RBC (*10^12^/L)	3.65 (3.27–4.04)	3.64 (3.26–4.02)	3.68 (3.34–4.08)	0.325^a^
	HB (g/L)	115 (103–126)	115 (102–126)	116 (104–127)	0.387^a^
	LYM%	27.30 (20.38–35.10)	27.20 (20.33–35.10)	28.35 (20.45–35.73)	0.439^a^
	ALT (U/L)	16 (12–22)	16 (12–22)	16 (13–24)	0.203^a^
	AST (U/L)	23 (19–28)	23 (19–28)	23 (19–27)	0.726^a^
	TBIL (umol/L)	8.70 (6.60–11.70)	8.60 (6.60–11.70)	8.85 (6.48–11.60)	0.624^a^
	DBIL (umol/L)	3.95 (3.20–5.10)	3.90 (3.20–5.10)	4.00 (3.10–5.20)	0.884^a^
	IBIL (umol/L)	4.60 (3.20–6.40)	4.50 (3.20–6.40)	4.90 (3.30–6.33)	0.528^a^
	GGT (U/L)	17 (12–27)	17 (12–27)	17 (12–31)	0.682^a^
	AKP (U/L)	69 (57–85)	70 (57–85)	69 (58–85)	0.984^a^
	Cr (umol/L)	82 (71–97)	82 (71–98)	81 (69–95)	0.138^a^
	BUN (mmol/L)	5.40 (4.40–6.40)	5.40 (4.40–6.50)	5.40 (4.20–6.30)	0.254^a^
	eGFR (ml/min)	85.35 (69.40–96.15)	85.40 (68.80–96.70)	85.30 (73.98–95.13)	0.490^a^

*Abbreviations: GIST* Gastrointestinal stromal tumor, *IM* Imatinib, *WBC* White blood cell, *PLT* Platelet count, *NEU*% Percentage of neutrophils, *RBC* Red blood cell count, *HB* Hemoglobin, *LYM*% Percentage of lymphocytes, *ALT* Alanine aminotransferase, *AST* Aspartate aminotransferase, *TBIL* Total bilirubin, *DBIL* Direct bilirubin, *IBIL* Indirect bilirubin, *GGT* Gamma-glutamyl transpeptidase, *AKP* Alkaline phosphatase, *Cr* Creatinine, *BUN* Urea nitrogen, *eGFR* Estimated glomerular filtration rate, *DOG-1* Gastrointestinal stromal tumor protein 1, *CD117* Cluster of differentiation 117, *CD34* Cell differentiation factor 34

^a^Mann–Whitney U-test; ^b^Pearson chi-square test; We calculated all P values as two-tailed; “Age at diagnosis” referred to the age at diagnosis of primary localized GIST; “Age at blood sampling” referred to the age at primary localized GIST patients took IM; Percentages might not always add up to exactly 100% as a result of rounding

**Table 2:** Comparison of baseline characteristics between “IM C_min_ ≤ 1100 ng/ml” and “IM C_min_ > 1100 ng/ml”

Categories	Variables	All	IM C_min_ ≤ 1100ng/ml	IM C_min_ > 1100ng/ml	*P*
		(***n*** = 890)	(***n*** = 278)	(***n*** = 612)	
Demographic information	Age at diagnosis (years)	56 (49–65)	55 (49–62)	58 (50–66)	0.006^a^
	Age at blood sampling (years)	61 (53–68)	59 (53–67)	62 (54–68)	0.004^a^
	Gender				< 0.001^b^
	Male	526 (59.10%)	212 (76.26%)	314 (51.31%)	
	Female	364 (40.90%)	66 (23.74%)	298 (48.69%)	
Treatment information	Surgical procedure				0.786^b^
	Gastrectomy	165 (18.54%)	53 (19.06%)	112 (18.30%)	
	Non-gastric operation	725 (81.46%)	225 (80.94%)	500 (81.70%)	
	Daily IM dose				0.010^b^
	≤ 200mg/d	28 (3.15%)	17 (6.12%)	11 (1.80%)	
	300mg/d	89 (10.00%)	27 (9.71%)	62 (10.13%)	
	400mg/d	679 (76.29%)	204 (73.38%)	475 (77.61%)	
	500mg/d	54 (6.07%)	20 (7.19%)	34 (5.56%)	
	≥ 600mg/d	40 (4.49%)	10 (3.60%)	30 (4.90%)	
Clinical information	DOG-1				0.788^b^
	Positive	859 (96.52%)	269 (96.76%)	590 (96.41%)	
	Negative	31 (3.48%)	9 (3.24%)	22 (3.59%)	
	CD117				0.639^b^
	Positive	878 (98.65%)	275 (98.92%)	603 (98.53%)	
	Negative	12 (1.35%)	3 (1.08%)	9 (1.47%)	
	CD34				0.138^b^
	Positive	795 (89.33%)	242 (87.05%)	553 (90.36%)	
	Negative	95 (10.67%)	36 (12.95%)	59 (9.64%)	
	Primary tumor site				0.163^b^
	Stomach	293 (32.92%)	91 (32.73%)	202 (33.01%)	
	Small intestine	352 (39.55%)	121 (43.53%)	231 (37.75%)	
	Colorectum	74 (8.31%)	16 (5.76%)	58 (9.48%)	
	Other	171 (19.21%)	50 (17.99%)	121 (19.77%)	
	Metastatic site				0.024^b^
	Liver	405 (45.51%)	142 (51.08%)	263 (42.97%)	
	Non-liver	485 (54.49%)	136 (48.92%)	349 (57.03%)	
Laboratory indicators	WBC (*10^9^/L)	4.32 (3.47–5.39)	4.33 (3.45–5.44)	4.31 (3.47–5.38)	0.812^a^
	PLT (*10^9^/L)	173 (136–220)	171 (129–217)	176 (137–220)	0.355^a^
	NEU%	61.25 (53.30–68.90)	59.75 (51.73–66.70)	62.30 (54.00–70.18)	< 0.001^a^
	RBC (*10^12^/L)	3.65 (3.27–4.04)	3.86 (3.52–4.30)	3.54 (3.18–3.91)	< 0.001^a^
	HB (g/L)	115 (103–126)	121 (109–134)	112 (101–123)	< 0.001^a^
	LYM%	27.30 (20.38–35.10)	29.65 (22.23–37.03)	26.50 (19.60–34.50)	< 0.001^a^
	ALT (U/L)	16 (12–22)	18 (13–26)	15 (12–21)	< 0.001^a^
	AST (U/L)	23 (19–28)	24 (19–29)	23 (19–27)	0.051^a^
	TBIL (umol/L)	8.70 (6.60–11.70)	8.90 (6.88–12.13)	8.50 (6.40–11.30)	0.026^a^
	DBIL (umol/L)	3.95 (3.20–5.10)	4.00 (3.30–5.40)	3.90 (3.10–5.00)	0.065^a^
	IBIL (umol/L)	4.60 (3.20–6.40)	4.65 (3.58–6.73)	4.50 (3.10–6.30)	0.026^a^
	GGT (U/L)	17 (12–27)	19 (14–31)	16 (11–25)	< 0.001^a^
	AKP (U/L)	69 (57–85)	70 (57–87)	69 (57–84)	0.774^a^
	Cr (umol/L)	82 (71–97)	85 (75–96)	81 (69–98)	0.038^a^
	BUN (mmol/L)	5.40 (4.40–6.40)	5.60 (4.50–6.60)	5.30 (4.20–6.30)	0.010^a^
	eGFR (ml/min)	85.35 (69.40–96.15)	86.55 (73.55–98.40)	85.00 (68.20–95.35)	0.033^a^

*Abbreviations*: *GIST* Gastrointestinal stromal tumor, IM Imatinib, WBC White blood cell, PLT Platelet count, NEU% Percentage of neutrophils, RBC Red blood cell count, HB Hemoglobin, LYM% Percentage of lymphocytes, ALT Alanine aminotransferase, AST Aspartate aminotransferase, TBIL Total bilirubin, DBIL Direct bilirubin, IBIL Indirect bilirubin, GGT Gamma-glutamyl transpeptidase, AKP Alkaline phosphatase, Cr Creatinine, BUN Urea nitrogen, eGFR Estimated glomerular filtration rate, DOG-1 Gastrointestinal stromal tumor protein 1, CD117 Cluster of differentiation 117, CD34 Cell differentiation factor 34

^a^Mann–Whitney U-test; ^b^Pearson chi-square test; We calculated all P values as two-tailed; “Age at diagnosis” referred to the age at diagnosis of primary localized GIST; “Age at blood sampling” referred to the age at primary localized GIST patients took IM; Percentages might not always add up to exactly 100% as a result of rounding

### Key variables

**Fig. 2:**
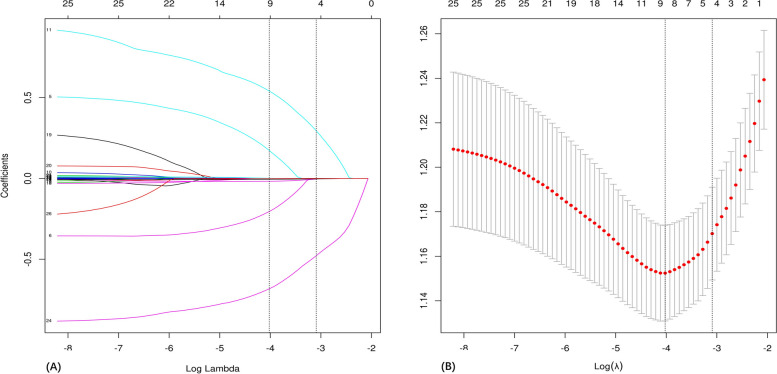
The processes of LASSO regression for screening variables. **A** The use of tenfold cross-validation to draw vertical lines at selected feature values. **B** The coefficient profiles of 26 feature variables were obtained from the log (λ) sequence in the LASSO model. Vertical dotted lines are placed at the minimal mean square error (λ = 0.018) and the standard error of the minimum distance (λ = 0.045). Abbreviations: LASSO, least absolute shrinkage and selection operator

In the training and validation sets, the 26 candidates underwent a tenfold cross-validation LASSO regression analysis (Fig. 2A and B). The results showed that the optimal parameter λ (λ = 0.018) in the LASSO regression analysis with the smallest mean square error, which reduced the 26 candidates to 9 feature variables, including daily IM dose, Metastatic site, Gender, PLT, NEU%, RBC, HB, LYM%, and age at diagnosis. To address potential confounding factors, the binary LR was used to analyze the above 9 feature variables via the forward-stepwise method. Finally, only daily IM dose, Metastatic site, Gender, PLT, NEU%, and RBC were determined as key variables (*p* < 0.05), as shown in Table 3.

**Table 3:** Forward stepwise binary logistic regression analysis

Variable	R	SE	OR (95% CI)	*p*
RBC (*10^12^/L)	-1.148	0.174	0.317 (0.226–0.446)	< 0.001
NEU%	0.026	0.008	1.027 (1.010–1.043)	0.001
PLT	0.003	0.001	1.003 (1.000–1.006)	0.029
Gender (“Female” for reference)	-0.691	0.207	0.501 (0.334–0.752)	< 0.001
Metastatic site (“Liver” for reference)	0.407	0.181	1.502 (1.054–2.140)	0.024
Daily IM dose (“ ≤ 200mg/d” for reference)				0.007
300mg/d	1.158	0.596	3.185 (0.991–10.235)	
400mg/d	1.779	0.546	5.923 (2.030–17.281)	
500mg/d	1.556	0.631	4.740 (1.375–16.341)	
≥ 600mg/d	2.014	0.732	7.490 (1.784–31.445)	

Through forward stepwise binary logistic regression analysis, variables such as HB, LYM%, and age at diagnosis are excluded

*Abbreviations*: *NEU%* Percentage of neutrophils, *HB* Hemoglobin, *RBC* Red blood cell count, *LYM% *Percentage of lymphocytes, *PLT* Platelet count, *R* Regression coefficient, *SE* Standard error, *OR* Odds ratio

### The best model building

**Fig. 3:**
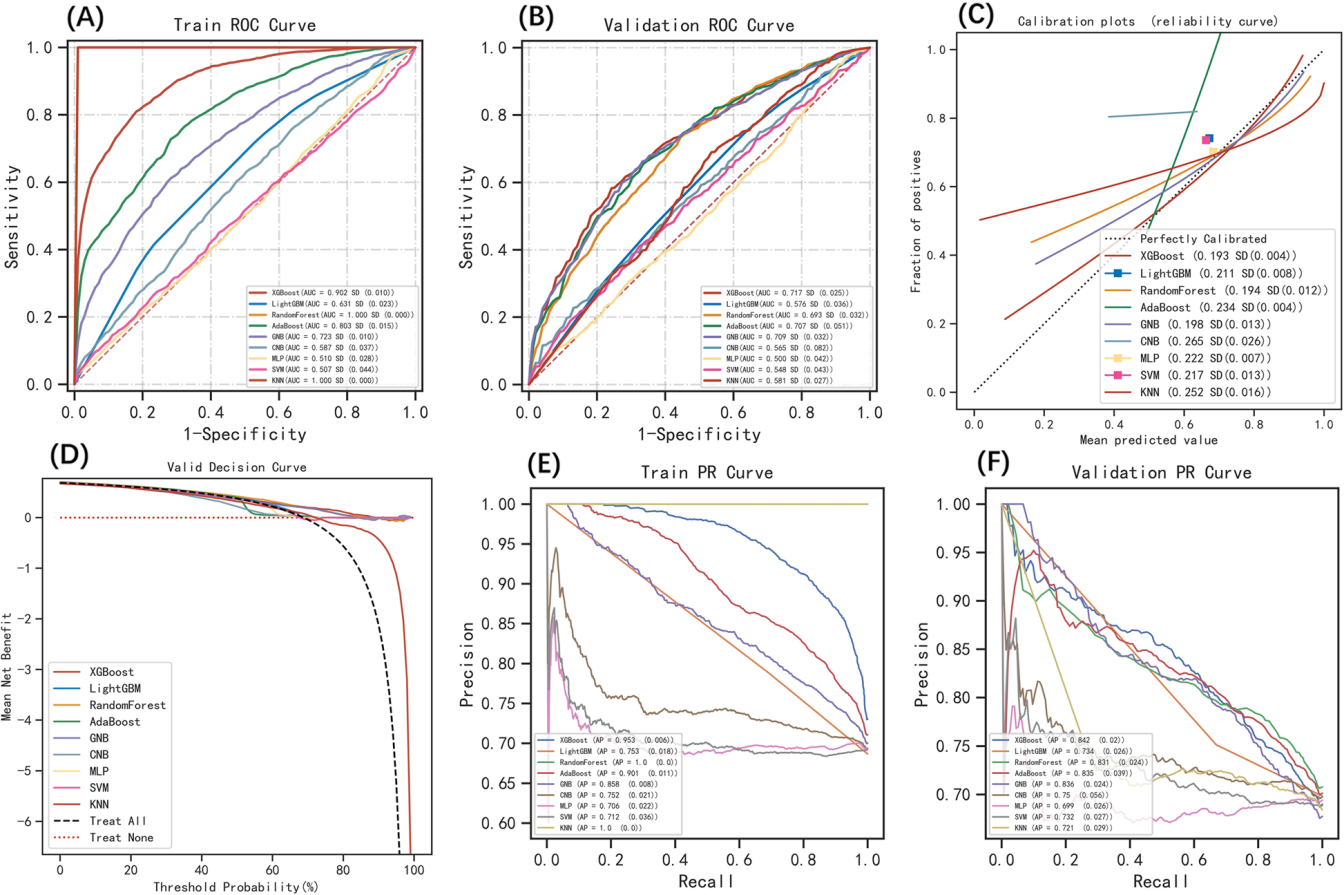
In training and validation sets, multiple ML classification models are integrated for analysis. **A** ROC curves evaluated the classification accuracy of the 9 models in the train set: XGBoost (AUROC = 0.902), LightGBM (AUROC = 0.631), RF (AUROC = 1.000), AdaBoost (AUROC = 0.803), GNB (AUROC = 0.723), CNB (AUROC = 0.587), MLP (AUROC = 0.510), SVM (AUROC = 0.507), and KNN (AUROC = 1.000). **B** ROC curves evaluated the classification accuracy of the 9 models in the validation set: XGBoost (AUROC = 0.717), LightGBM (AUROC = 0.576), RF (AUROC = 0.693), AdaBoost (AUROC = 0.707), GNB (AUROC = 0.709), CNB (AUROC = 0.565), MLP (AUROC = 0.500), SVM (AUROC = 0.548), and KNN (AUROC = 0.581). **C** The calibration curve for different models in the validation set, the abscissa represents the average prediction probability, the ordinate represents the actual probability of the event, the dashed diagonal is the reference line, and the other smooth solid lines represent the different model fitting lines. Brier scores evaluated the calibration of the 9 models: XGBoost (Brier score = 0.193), LightGBM (Brier score = 0.211), RF (Brier score = 0.194), AdaBoost (Brier score = 0.234), GNB (Brier score = 0.198), CNB (Brier score = 0.265), MLP (Brier score = 0.222), SVM (Brier score = 0.217), and KNN (Brier score = 0.252). **D** The decision curve for different models in the validation set. The solid lines represent different models. **E** The AUPR curve for different models in the training set, the y‐axis is precision and the x‐axis is recall. AUPR evaluated the overall performance of the 9 models in the train set: XGBoost (AUPR = 0.953), LightGBM (AUPR = 0.753), RF (AUPR = 1.000), AdaBoost (AUPR = 0.901), GNB (AUPR = 0.858), CNB (AUPR = 0.752), MLP (AUPR = 0.706), SVM (AUPR = 0.712), and KNN (AUPR = 1.000). **F** The AUPR curve for different models in the validation set, the y‐axis is precision and the x‐axis is recall. AUPR evaluated the overall performance of the 9 models in the validation set: XGBoost (AUPR = 0.842), LightGBM (AUPR = 0.734), RF (AUPR = 0.831), AdaBoost (AUPR = 0.835), GNB (AUPR = 0.836), CNB (AUPR = 0.750), MLP (AUPR = 0.699), SVM (AUPR = 0.732), and KNN (AUPR = 0.721). Abbreviations: XGBoost, Extreme Gradient Boosting; LightGBM, Light Gradient Boosting Machine; RF, Random Forest; GNB, Gaussian Naive Bayes; CNB, Complement Naive Bayes; MLP, Multilayer Perceptron; SVM, Support Vector Machine; KNN, K-Nearest Neighbour; AdaBoost, Adaptive Boost; AUROC, area under the receiver-operating characteristic curve; ROC, receiver operating characteristic; AUPR, area under the precision-recall curve; PR, precision-recall curve; AUC, area under curve

Following the identification of these six key variables, XGBoost, LightGBM, RF, GNB, CNB, MLP, SVM, KNN, and AdaBoost were trained and applied the resampling method by resampling 10 times. As shown in Fig. 3A and B, RF and KNN (ranked according to AUROC) had the best performance in the training set, but XGBoost (ranked according to AUROC) had the largest AUROC and shortest SD in the validation set, indicating the best stability of this model. When the Brier scores for the nine aforementioned ML models were compared, that of XGBoost was the lowest, indicating that its prediction calibration was the best (Brier scores = 0.193, Fig. 3C). XGBoost model reveals the largest area under the decision curve, indicating a better clinical utility than other models (Fig. 3D). The PR curve is sensitive to data imbalance, and it changes dramatically as the ratio of positive to negative samples changes [25]. As we know, the larger the AUPR, the higher the average precision of the model. Although in the training set, RF and KNN had the largest value of AUPR (Fig. 3E), in the validation set, the PR curve area of the XGBoost model was the largest (AUPR = 0.842) (Fig. 3F). Based on the above results, the XGBoost model may be the optimal model choice for this dataset, rather than the RF and KNN models, which may overfit data.

### The best model evaluation

**Fig. 4:**
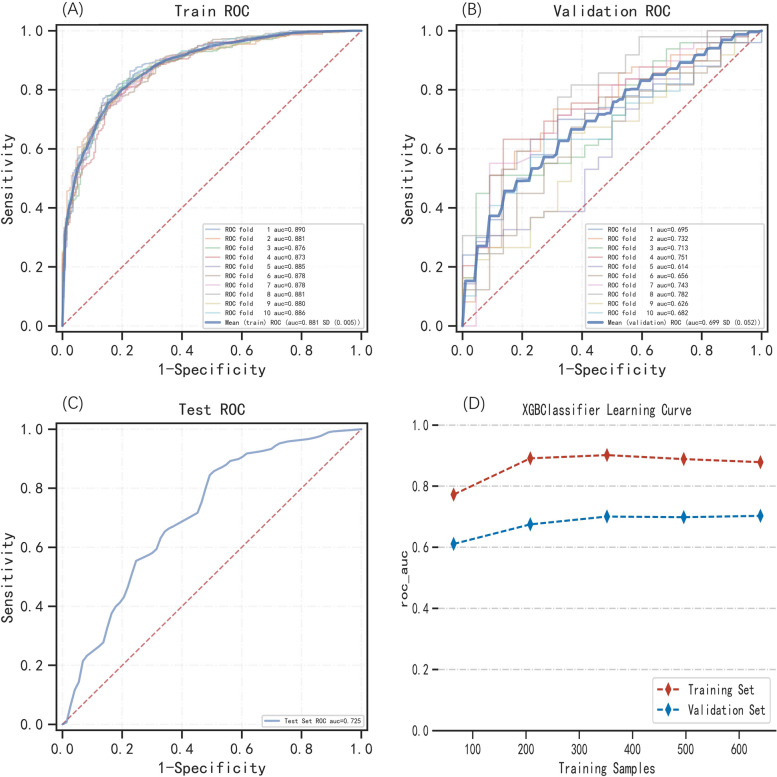
The performance of the XGBoost model was evaluated by tenfold cross‐validation in the training set and internal validation in the test set. **A** The mean AUROC for the XGBoost model in the training set (AUROC = 0.881). **B** The mean AUROC for the XGBoost model in the validation set (AUROC = 0.699). **C** The AUROC for the XGBoost model in the test set (AUROC = 0.725). **D** In the learning curve, the red dashed line represents the training set and the blue dashed line represents the validation set. Abbreviations: XGBoost, Extreme Gradient Boosting; AUROC, area under the receiver-operating characteristic curve; ROC, receiver operating characteristic; AUC, area under curve

The XGBoost ML algorithm analysis and tenfold cross-validation were performed on the dataset. According to the findings, the training set's average AUROC was 0.881 (0.873–0.890, Fig. 4A), the validation set's average AUROC was 0.699 (0.614–0.782, Fig. 4B), and the testing set's AUROC was 0.725 (Fig. 4C). If the validation set's AUROC is lower than the test set's, the model fitting could be considered successful, indicating that the model has good generalization [26]. Meanwhile, as shown in Fig. 4D, the learning curve revealed that the training and validation sets were well-fitting and stable [26–28]. As a result, the above results revealed that the XGBoost algorithm might be employed for this dataset's classification modeling purpose.

**Fig. 5:**
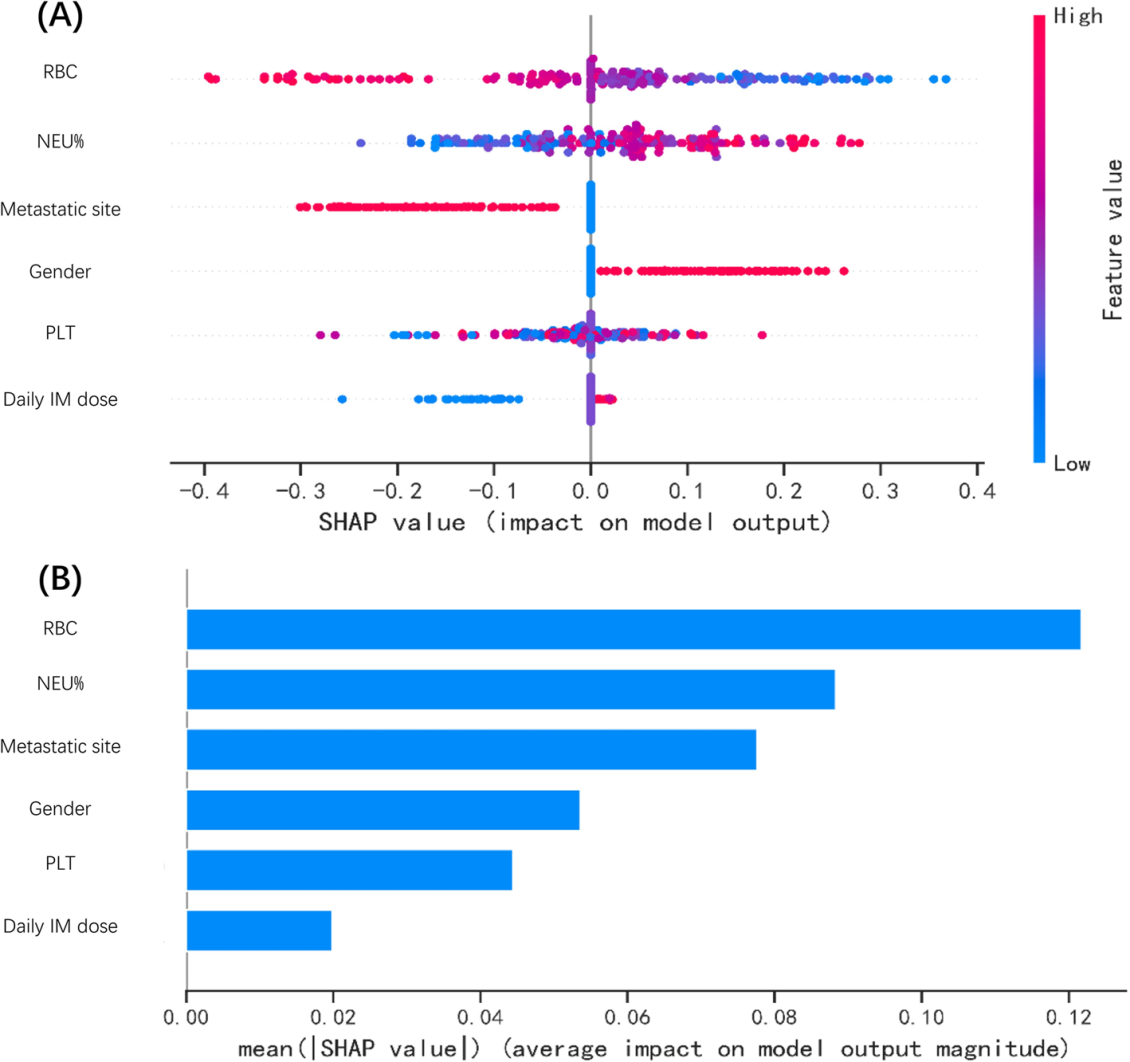
SHAP summary graph of the XGboost model. **A** This diagram describes the dot estimation on the model output of the XGBoost model. Each dot represents an individual patient from the dataset. The colors represent the feature value, red represents the higher SHAP value of specific features, and blue represents the lower SHAP value of specific features. **B** Average absolute impact of variables on the output value of the XGBoost model (ranked in descending order of feature importance). Abbreviations: XGBoost, Extreme Gradient Boosting; SHAP, Shapley Additive Explanations; RBC, red blood cell count; NEU%, percentage of neutrophils; PLT, platelet count

The SHAP analyzes the entire test set, visually explaining the impact of six key variables on the XGBoost model. Furthermore, in the SHAP analysis of the XGBoost model, the color represents the value of the variable, red pixels symbolize positive SHAP values enhancing class likelihood, while blue pixels denote negative SHAP values reducing class probability (Fig. 5A). The bar chart shows the relationship between the magnitude of the feature value and the predicted impact (Fig. 5B).

## Discussion

Demetri et al. [8] previously reported that advanced GIST patients with IM C_min_ < 1100 ng/mL had a shorter progression-free time in 2009. Although there is still controversy about the optimal cut-off value for IM C_min_, in clinical practice, "1100 ng /ml" has become a common reference value for monitoring IM C_min_ in outpatients. In this study, we thus used 1100 ng/ml as the cutoff value and converted IM C_min_, a continuous variable, into a binary variable. We compared nine common ML algorithms. The optimal ML model was selected using AUROC, DCA, Brier Scores, and AUPR. Finally, the XGBoost model was selected as the best model for analysis, internally validated, and proved to have good classification.

The relationships between the label variable (IM C_min_) and feature variables were assessed using LASSO regression and LR. Six key variables (daily IM dose, metastatic site, gender, PLT, NEU%, and RBC) were screened out, which were easy to obtain, and also were the key variables in constructing the XGBoost model in this study. Interestingly, except for PLT, these key variables reached also statistical significance in Table 2. Some studies believe that IM is mainly metabolized in the liver [29, 30]. Therefore, before our data analysis, Laboratory indicators related to liver function examination were expected to be key and important features in constructing the classification model. However, to our surprise, the features that were finally screened by parameters did not include laboratory indicators related to liver function examination. We consider that the reason for this phenomenon may be that our outcome variables are binary, whereas the outcome variable in the previous study was continuous. This difference may lead to the exclusion of laboratory indicators related to liver function examination in the final selection of variables.

IM C_min_ was shown to be higher in females than in males in several studies, and researchers believed the difference could be attributable to differences in body weight or medication adherence between genders [31, 32]. The existence of liver metastases may result in more changes and increased exposure to IM, which may cause higher in IM C_min_ [33]. A previous study (Eechoute, 2012) found that IM clearance was expected to decrease by 3.8% for every 100 cm^3^ increase in liver metastatic volume [29]. Previous studies had reported the relationship between daily IM dose and IM C_min_ [17, 18, 34], the TDM for IM provided a reference for the adjustment of IM dosage, which added to the utility of TDM in the management of patients with GISTs [12]. It is worth noting that no foreign studies have previously reported the effect of RBC on imatinib clearance, but a recent domestic study confirmed that RBC had a significant effect on the clearance of IM [35], which may be due to ethnic differences between domestic and foreign study populations. Thrombocytopenia and neutropenia are common side effects of IM-targeted therapy [36], which may be why PLT and NEU% are key variables in constructing the model.

IM is an anti-cancer drug administered primarily to outpatients because blood samples are not always available at the end of the administration interval. Thus, IM C_min_ is the most widely used pharmacokinetic proxy for predicting clinical outcomes [7, 37], and C_min_ is naturally used as a focus for TDM [38]. TDM for IM may reassure patients and physicians about full exposure to the drug and improve long-term adherence to this chronic treatment, which may be a promising approach for fine-tuning the IM dosage for better tolerability and optimal clinical outcomes in patients with GISTs [7, 37]. It is widely known that high IM C_min_ increases the risk of adverse effects and toxicity, which can reduce medication adherence rates and quality of life. Therefore, it is crucial for patients with GISTs to frequently undergo TDM of IM [34]. However, most hospitals are unable to monitor IM C_min_ because they do not have the equipment to do so, which makes the IM C_min_ classification model valuable for clinical application.

Precision therapy stands as a primary use of ML, offering patients customized medical services including individualized dosage modification, plasma concentration prediction, and prediction of negative drug reactions [13, 39, 40]. In clinical practice, 1100 ng/mL is often used as the reference value, combined with the patient's drug tolerance and the change in CT tumor lesions, to evaluate the drug efficacy and adjust the drug dosage [41]. For example, patients with IM C_min_ less than 1100 ng/ml (which is predicted by the XGBoost model), where tumor progression is defined by imaging and/or symptomatic progression, could be encouraged to appropriately increase the doses. By the same token, patients with IM C_min_ greater than 1100 ng/ml, as predicted by the XGBoost model, would experience serious adverse drug reactions and could be encouraged to appropriately reduce their doses. Using the above two examples, we know that using machine-learning methods to detect blood drug concentrations could help some hospitals without the TDM platform reduce their healthcare burden. For some hospitals with the TDM platform, sometimes, the ML model is more often used to streamline IM C_min_ monitoring rather than completely replace TDM.

A model developed by Gotta in 2012 showed that the Bayesian MAP-ρ method, which considered the correlation between pharmacokinetic parameters, could predict IM C_min_ with an unbiased accuracy of ± 30.7% [42]. 

The difference between this study and the above study mainly lies in the study population, study design, and study variables. First, IM C_min_ measured in the adjuvant setting is excluded. Second, the classification model includes six feature variables that are easily accessible during usual treatment. This advantage enables the model to be generalized and applied well. Finally, to our knowledge, this is the first study to develop and internally validate a classification model for IM C_min_ that has high predictive performance, which, combines with Demetri's study [8], may aid in prognostic prediction in patients with advanced GISTs. Therefore, in the future, we plan to further establish a web application that is easy to use based on the presented XGBoost classification model, which could then be used as a real-time clinical decision support tool through self-learning and optimization and aid in personalized IM dose adjustment.

Although the new model has good predictive performance, there are still some considerable limitations to this study. First, the limited number of samples available may reduce the performance of the XGBoost model. Second, given its nature as a retrospective, single-center research with an extended duration, it faces all the constraints typical of retrospective studies. For instance, the lack of pharmacokinetic parameters and body surface area data, incomplete laboratory indicators, and fluctuations in blood collection time points may all affect IM C_min_. Therefore, in this study, the classification prediction of IM C_min_ is the next best thing, rather than the specific value prediction, which is continuous. For this reason, our current model is more of a reference than a complete replacement for TDM. Third, while our classification model has been internally validated, additional prospective validation should be performed in future studies, or a wholly external dataset should be employed for external validation to improve the generalization ability of this model. Finally, as several works of literature suggest polymorphism effects on exposure and drug-drug interaction via CYP3A [30, 43–45], changes in C_min_ estimation could be suspected, but those indicators are not included in this research. In future work and research, we will make efforts to make up for the above deficiencies and establish a new model, and the result variable of this model is a continuous value, to help some hospitals without the TDM platform reduce their healthcare burden, or even replace TDM.

## Conclusion

We developed and validated ML models for individualized classification of IM C_min_ tailored to patients with advanced GISTs from China by utilizing readily available baseline information and assay indices, which were easy to obtain. This XGBoost model showed good classification performance and had good clinical application value.

## Data Availability

The datasets generated and analyzed during the current study are not publicly available due to a total of 890 IM Cmin samples included in the study from 212 patients with advanced GISTs have been deposited in the “Weinichangzai” database, but are available from the corresponding author on reasonable request.

## Abbreviations

GIST: Gastrointestinal stromal tumor
IM: Imatinib
C_min_: Plasma trough concentration
AUROC: Area under the receiver-operating characteristic curve
ROC: Receiver operating characteristic
AUPR: Area under the precision-recall curve
PR: Precision-recall curve
AUC: Area under curve
SHAP: Shapley additive explanations
KIT: Kinase growth factor receptor proto-oncogene
TKI: Tyrosine kinase inhibitor
TDM: Therapeutic drug monitoring
ML: Machine learning
CMU: Chongqing Medical University
WBC: Blood cell count
PLT: Platelet count
NEU%: Percentage of neutrophils
RBC: Red blood cell count
HB: Hemoglobin
LYM%: Percentage of lymphocytes
ALT: Alanine aminotransferase
AST: Aspartate aminotransferase
TBIL: Total bilirubin
DBIL: Direct bilirubin
IBIL: Indirect bilirubin
GGT: Gamma-glutamyl transpeptidase
AKP: Alkaline phosphatase
Cr: Creatinine
BUN: Urea nitrogen
eGFR: Estimated glomerular filtration rate
LASSO: Least absolute shrinkage and selection operator
LR: Logistic regression
XGBoost: Extreme Gradient Boosting
LightGBM: Light Gradient Boosting Machine
RF: Random forest
GNB: Gaussian Naive Bayes
CNB: Complement Naive Bayes
MLP: Multilayer Perceptron
SVM: Support vector machine
KNN: K-nearest neighbour
AdaBoost: Adaptive boost
IQR: Interquartile range
SD: Standard deviation
DOG-1: Gastrointestinal stromal tumor protein 1
CD117: Cluster of differentiation 117
CD34: Cell differentiation factor 34
R: Regression coefficient
SE: Standard error
OR: Odds ratio
